# Size Matters: Ultra-small and Filterable Microorganisms in the Environment

**DOI:** 10.1264/jsme2.ME20025

**Published:** 2020-06-03

**Authors:** Ryosuke Nakai

**Affiliations:** 1 Applied Molecular Microbiology Research Group, Bioproduction Research Institute, National Institute of Advanced Industrial Science and Technology (AIST), 2–17–2–1, Tsukisamu-Higashi, Sapporo, 062–8517, Japan

**Keywords:** filterable microorganisms, ultramicrocells, ultramicrobacteria, candidate phyla radiation, minimal cell

## Abstract

Ultra-small microorganisms are ubiquitous in Earth’s environments. Ultramicrobacteria, which are defined as having a cell volume of <0.1 μm^3^, are often numerically dominant in aqueous environments. Cultivated representatives among these bacteria, such as members of the marine SAR11 clade (*e.g.*, “*Candidatus* Pelagibacter ubique”) and freshwater *Actinobacteria* and *Betaproteobacteria*, possess highly streamlined, small genomes and unique ecophysiological traits. Many ultramicrobacteria may pass through a 0.2-μm-pore-sized filter, which is commonly used for filter sterilization in various fields and processes. Cultivation efforts focusing on filterable small microorganisms revealed that filtered fractions contained not only ultramicrocells (*i.e.*, miniaturized cells because of external factors) and ultramicrobacteria, but also slender filamentous bacteria sometimes with pleomorphic cells, including a special reference to members of *Oligoflexia*, the eighth class of the phylum *Proteobacteria*. Furthermore, the advent of culture-independent “omics” approaches to filterable microorganisms yielded the existence of candidate phyla radiation (CPR) bacteria (also referred to as “*Ca.* Patescibacteria”) and ultra-small members of DPANN (an acronym of the names of the first phyla included in this superphyla) archaea. Notably, certain groups in CPR and DPANN are predicted to have minimal or few biosynthetic capacities, as reflected by their extremely small genome sizes, or possess no known function. Therefore, filtered fractions contain a greater variety and complexity of microorganisms than previously expected. This review summarizes the broad diversity of overlooked filterable agents remaining in “sterile” (<0.2-μm filtered) environmental samples.

How small may actual organisms be? This question has long fascinated scientists in various fields. Prokaryotic microorganisms (*Archaea* and *Bacteria*) constitute the smallest life forms. Bacterial cells range in volume from ultramicrobacteria (UMB; <0.1 μm^3^; Duda *et al.*, 2012) to the typical bacterium *Escherichia coli* (1.6 μm^3^; Moore, 1999) and the giant bacterium *Epulopiscium fishelsoni* (3.0×10^6^ μm^3^; Schulz and Jørgensen, 2001; note that the cells of *Thiomargarita namibiensis* are larger [2.2×10^8^ μm^3^], but are occupied by a liquid vacuole, that is, they do not have large cytoplasmic bodies; Schulz *et al.*, 1999). Thus, bacteria exhibit cell-size plasticity by varying cell volume by more than seven orders of magnitude in different species. UMB may pass through membrane filters down to 0.2-μm-pore-size, which is commonly used for filter sterilization in research laboratories as well as in medical, food, and industrial processes (Levy and Jornitz, 2006). In fact, efforts to culture microorganisms remaining in the 0.2-μm filtrate (hereafter called filterable microorganisms) of environmental samples have yielded diverse UMB members. The several isolates were affiliated with unique lineages, such as cosmopolitan freshwater *Actinobacteria* and *Betaproteobacteria* (Hahn, 2003; Hahn *et al.*, 2003) as well as the candidate phylum termite group 1 (TG1) described as *Elusimicrobia* (Geissinger *et al.*, 2009). The existence of UMB has expanded our knowledge of microbial life at the lower size limit.

In the last five years, filterable microorganisms have been attracting increasing interest with the discovery of other ultra-small members: the candidate phyla radiation (CPR) bacteria, also referred to as “*Candidatus* Patescibacteria” (hereafter described as CPR/Patescibacteria; Rinke *et al.*, 2013; Brown *et al.*, 2015), and some members of DPANN (an acronym of the names of the first phyla included in this superphyla, “*Ca.* Diapherotrites”, “*Ca.* Parvarchaeota”, “*Ca.* Aenigmarchaeota”, *Nanoarchaeota*, and “*Ca.* Nanohalorchaeota”; Rinke *et al.*, 2013; Dombrowski *et al.*, 2019). Several CPR members have an extremely small cell volume (approximately 0.01 μm^3^) that was unveiled by cryo-transmission electron microscopy imaging (Luef *et al.*, 2015). Moreover, the emergence of these ultra-small prokaryotes has re-opened debate on the tree of life (Hug *et al.*, 2016; Parks *et al.*, 2018; Zhu *et al.*, 2019). These members are ubiquitous in the environment and recent studies have provided insights into their contribution to the material cycle (*e.g.*, carbon and nitrogen cycles; Danczak *et al.*, 2017; Lannes *et al.*, 2019). This review focuses on the phylogenetic diversity and complexity of filterable microorganisms in natural systems, with specific references to UMB and pleomorphic bacteria. Other reviews presented aspects of ultra-small microorganisms including CPR/Patescibacteria and DPANN members (*e.g.*, terminology, biogeography, genomic diversity, and metabolic variety; Duda *et al.*, 2012; Castelle *et al.*, 2018; Ghuneim *et al.*, 2018; Dombrowski *et al.*, 2019). In this review, archaea with a cell volume of <0.1 μm^3^ are specifically referred to as ultramicroarchaea (UMA) to distinguish them from UMB.

## Filterable microorganisms

To date, many studies have reported the presence of filterable microorganisms in various environments (mainly aqueous environments) including seawater (Haller *et al.*, 2000; Elsaied *et al.*, 2001; Lannes *et al.*, 2019; Obayashi and Suzuki, 2019), lake water (Hahn, 2003; Hahn *et al.*, 2003; Watanabe *et al.*, 2009; Fedotova *et al.*, 2012; Maejima *et al.*, 2018; Vigneron *et al.*, 2019), terrestrial aquifers (Miyoshi *et al.*, 2005; Luef *et al.*, 2015), glacier ice and the ice cover of lakes (Miteva and Brenchley, 2005; Kuhn *et al.*, 2014), deep-sea hydrothermal fluids (Naganuma *et al.*, 2007; Nakai *et al.*, 2011), and soil and sand (Nakai *et al.*, 2013). However, the use of membrane filters with a small pore size (approximately 0.2 μm) was traditionally recommended for the retention of bacteria in the field of marine microbial ecology in the 1960s (*e.g.*, Anderson and Heffernan, 1965) and is still widely practiced today in various fields. The existence of very small microorganisms has been well recognized since the 1980s. The term “ultramicrobacteria” was first used by Torrella and Morita (1981) to describe very small coccoid cell forms of <0.3 μm in diameter from seawater. MacDonell and Hood (1982) subsequently isolated and characterized viable filterable microorganisms potentially belonging to the genera *Vibrio*, *Aeromonas*, *Pseudomonas*, and *Alcaligenes* from estuarine waters. They concluded that these filterable microorganisms represented a state of dormancy for adaptation to low nutrient conditions and were not completely novel bacteria. Other studies also reported that external factors reduced cell sizes, such as *Staphylococcus aureus* and *Pseudomonas syringae* (~50% reduction in size as described in Table 1; Watson *et al.*, 1998; Monier and Lindow, 2003). Therefore, the cells of miniaturized microorganisms need to be distinguished from true UMB and are described in this review as “ultramicrocells”, which has the synonyms dwarf cells and midget cells, according to Duda *et al.* (2012). Schut *et al.* (1997) and Duda *et al.* (2012) subsequently defined a cell volume index of <0.1 μm^3^ as being characteristic of true UMB.

**Fig. 1.**
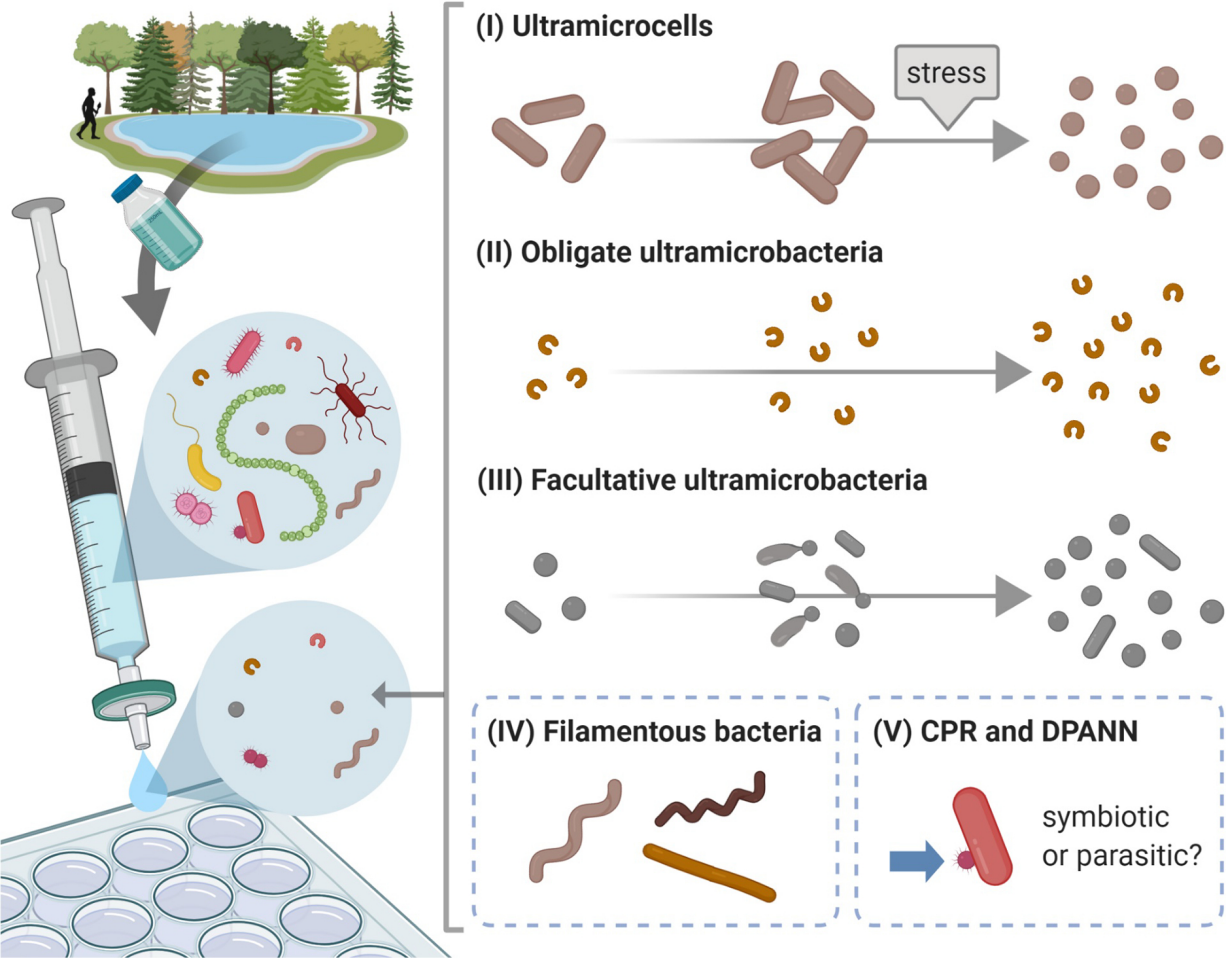
Diagram showing filterable microorganisms in the environment. (I) ultramicrocells; (II) obligate ultramicrobacteria; (III) facultative ultramicrobacteria; (IV) slender filamentous bacteria; (V) ultra-small members of CPR bacteria (also referred to as “*Candidatus* Patescibacteria”) and DPANN archaea indicated by the arrow in this Figure. See details in the text. This figure was created with BioRender (https://biorender.com/).

Based on previous studies, filterable microorganisms have been classified into five groups (Fig. 1): (I) ultramicrocells that are miniaturized microorganisms because of external factors (*e.g.*, environmental stress) as described above; (II) obligate UMB that maintain small cell volumes (<0.1‍ ‍μm^3^) regardless of their growth conditions; (III) facultative UMB that contain a small proportion of larger cells with a cell volume >0.1 μm^3^ (note that the definitions of the terms “obligate” and “facultative” UMB follow those of Duda *et al.* [2012]); (IV) slender filamentous bacteria; and (V) ultra-small members among CPR/Patescibacteria bacteria and DPANN archaea. In contrast to UMB strains, the cell shapes and morphological characteristics of members in group V are largely unknown under different environmental or culture conditions because all of the members of CPR and DPANN are uncultivated, with a few exceptions of members belonging to the phyla “*Ca.* Saccharibacteria” (former TM7) and *Nanoarchaeota* (*e.g.*, Huber *et al.*, 2002; He *et al.*, 2015). Incidentally, the groups presented in this review do not include filterable cell-wall-less mycoplasmas as well as “nanobacteria” or “nannobacteria” as microfossils, which are often referred to in geological literature (Folk, 1999), or as calcium carbonate nanoparticles in the human body, as reported in medical literature (Martel and Young, 2008). Representative cases of groups II to V are described below and Table 1 shows a summarized list.

## Obligate UMB

Obligate UMB are often reported from aqueous environments. One of the most prominent representatives is “*Candidatus* Pelagibacter ubique” HTCC1062, which is a SAR11 clade bacterium that is ubiquitous in marine environments. Previous studies found that SAR11 members consistently dominated ribosomal RNA gene clone libraries derived from seawater DNA and estimated their global population size as 2.4×10^28^ cells—approximately 25% of all prokaryotic cells—in oceans (Giovannoni *et al.*, 1990; Morris *et al.*, 2002). Despite their ubiquitous and abundant presence, it was not possible to isolate them. However, the first cultivated strain HTCC1062 was established in 2002 using a high-throughput dilution-to-extinction culturing (HTC) technique (Rappé *et al.*, 2002). This HTC technique involves cultivation with serial dilutions of natural seawater samples into very low nutrient media (Connon and Giovannoni, 2002). The cell volume (approximately 0.01‍ ‍μm^3^) of “*Ca.* P. ubique” was reported as one of the smallest free-living cells known. Subsequent studies characterized the SAR11 clade with the small, streamlined genomes (<1.5‍ ‍Mbp) described below, an unusual mode of glycine auxotrophy, a light-dependent proton pump known as proteorhodopsin, and the ability to utilize various one-carbon compounds (reviewed in Tripp, 2013; Giovannoni, 2017). The SAR11 clade is highly divergent with multiple ecotypes and has freshwater members known as LD12 classified in SAR11 subclade IIIb (Grote *et al.*, 2012). An LD12 cultivated representative, “*Ca.* Fonsibacter ubiquis” strain LSUCC0530, was subsequently established (Henson *et al.*, 2018), and its genomic characteristics promoted the hypothesis that gene losses for osmolyte uptake were related to the evolutionary transition, or metabolic tuning, of freshwater SAR11 (LD12) from a salt to freshwater habitat.

Another marine ultramicrobacterium, *Sphingopyxis alaskensis* (formerly known as *Sphingomonas alaskensis*) RB2256 was intensively investigated before the study of the SAR11 clade (*e.g.*, Eguchi *et al.*, 1996; Schut *et al.*, 1997). This strain was also characterized as an obligate UMB (Duda *et al.*, 2012). When the cultivation of this strain transitioned from low-carbon to highly-enriched media, the cell volume of *S. alaskensis* remained at <0.1 μm^3^ in most media; however, larger elongated cells, not UMB cells, were observed in trypticase soy agar medium (Vancanneyt *et al.*, 2001). Furthermore, this strain possesses a larger genome of 3.3‍ ‍Mb (DDBJ/ENA/GenBank accession no. CP000356) than other UMB (Table 1).

**Fig. 2.**
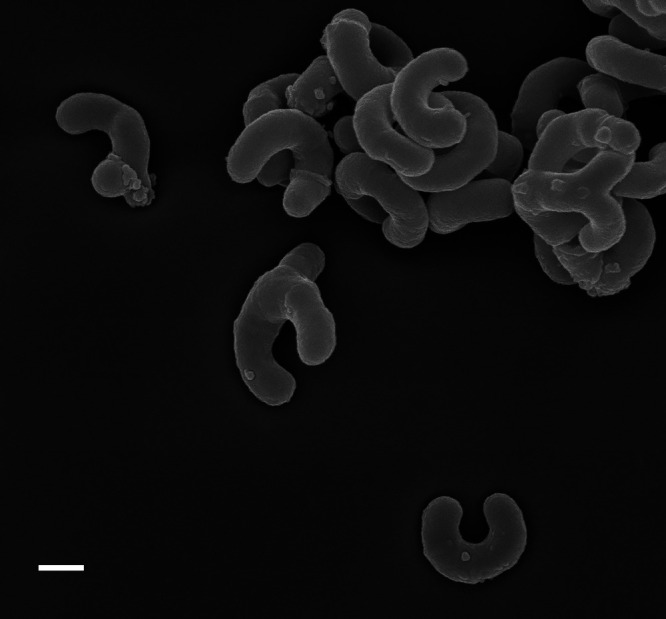
Scanning electron micrograph of c-shaped cells of *Aurantimicrobium minutum* KNC^T^. Cells were cultured in organic NSY (nutrient broth, soytone, and yeast extract; Hahn *et al.*, 2004) medium for two weeks. Scale bar: 200 nm. This micrograph is an unpublished figure from the author; other micrographs of this species are shown in Nakai *et al.* (2013, 2015).

Other prominent representatives of obligate UMB are freshwater actinobacterial strains. Typically, actinobacteria are among the numerically dominant groups in freshwater and their cells are found in smaller size fractions (Glöckner *et al.*, 2000; Sekar *et al.*, 2003). Hahn *et al.* (2003) first isolated nine filterable UMB of the class *Actinobacteria* from freshwater habitats and newly described a novel phylogenetic cluster (Luna cluster). This isolation was achieved by the “filtration-acclimatization” method of filter separation combined with an acclimatization procedure, which is a stepwise transition from low substrate conditions to artificial culture conditions. The important features of Luna cluster strains are their wide distribution in freshwater systems (Hahn and Pöckl, 2005) and their small cell sizes are stable and maintained in nutrient-rich media (Hahn *et al.*, 2003). Our group also isolated an ultamicrosize actinobacterium related to Luna strains from river water in Japan and named it *Aurantimicrobium minutum* KNC^T^ (Fig. 2; Nakai *et al.*, 2015). This strain showed high 16S rRNA gene sequence similarity (>99%) to strains isolated from freshwater systems in other places in Japan as well as in Austria, Australia, China, Nicaragua, and Uganda (accession nos. AB278121, AB599783, AJ507461, AJ507467, AJ565412, AJ565413, and AJ630367), suggesting its cosmopolitan distribution in freshwater.

The other freshwater bacterium belonging to the Luna cluster, *Rhodoluna lacicola* MWH-Ta8^T^, was also described as an obligate UMB (Hahn *et al.*, 2014); an additional three *Rhodoluna* strains smaller than *R. lacicola* were subsequently reported (Pitt *et al.*, 2019). From an eco-physiological point of view, the genomes of freshwater actinobacteria possess rhodopsin photosystems (Neuenschwander *et al.*, 2018), while *R. lacicola* has an unconventional proton-pumping rhodopsin that requires external supplementation with the cofactor retinal (Keffer *et al.*, 2015). The underlying cause is considered to be an inability to biosynthesize the cofactor (Neuenschwander *et al.*, 2018), suggesting that *R. lacicola* obtains retinal from the surrounding environment. One potential source in freshwater appears to be retinoids produced and released by cyanobacteria (Ruch *et al.*, 2005; Wu *et al.*, 2013).

Freshwater actinobacteria, including UMB strains, were previously shown to be phylogenetically diverse and subsequent studies yielded nine lineages (acI, acTH1, acSTL, Luna1, acIII, Luna3, acTH2, acIV, and acV; Newton *et al.*, 2011). Among these lineages, acI containing multiple tribes is considered to be the most successful and ubiquitous group in the environment (Zwart *et al.*, 2002; Warnecke *et al.*, 2004; Kang *et al.*, 2017), although pure cultures had not been established despite various cultivation trials. However, Kim *et al.* (2019) recently reported the first two pure acI cultures with very small sizes (volume, 0.04–0.06 μm^3^; Table 1), which are assumed to be obligate UMB. A key factor for their growth was the supplementation of a “helper” catalase, an enzyme that degrades hydrogen peroxide (H_2_O_2_), to the culture medium. Previous studies showed that H_2_O_2_ generated in medium affected the culture efficiency of microorganisms sensitive to oxidative stress (Kawasaki and Kamagata, 2017) and that the growth of the cyanobacterium *Prochlorococcus* was promoted by the presence of H_2_O_2_-scavenging microbes (Morris *et al.*, 2011). These findings demonstrated that a catalase-supplemented cultivation strategy may facilitate the successful isolation of previously uncultured freshwater UMB.

Freshwater habitats also harbor another obligate UMB belonging to the genus *Polynucleobacter* in the class *Betaproteobacteria*. Similar to some actinobacteria described earlier, UMB members of this genus also showed a cosmopolitan distribution in freshwater systems (Hahn, 2003). The relative abundance of the subspecies named PnecC was high, ranging between <1% and 67% (average 14.5%) of total bacterial numbers, in more than 130 lakes studied in Central Europe, as assessed by fluorescent *in situ* hybridization (Jezberová *et al.*, 2010). Culture experiments and genomic characterization suggested that PnecC bacteria in nature can utilize low-molecular-weight products derived from photooxidation and/or the direct enzymatic cleavage of high-molecular-weight substrates, such as humic substances (Watanabe *et al.*, 2009; Hahn *et al.*, 2012). Certain PnecC strains sharing ≥99% similarity in 16S rRNA gene sequences differed in their ecophysiological and genomic features (*e.g.*, the presence/absence of iron transporter genes), suggesting cryptic diversity among the abundant lineage not covered by 16S rRNA gene-based typing (Hahn *et al.*, 2016).

The obligate UMB inhabiting sea and freshwaters described above were characterized by minute cell sizes, but also small genome sizes (<2‍ ‍Mbp) with a low genomic guanine-cytosine (GC) content: this genome “streamlining” is considered to reflect an adaptation to nutrient-limited conditions (*e.g.*, SAR11 members; 1.16–1.46 Mb; Giovannoni *et al.*, 2005; Grote *et al.*, 2012; Henson *et al.*, 2018) (Table 1). This phenomenon of a reduced genome size with gene loss also indicates metabolic dependencies on co-existing microorganisms in nature, as described by the “Black Queen Hypothesis” (Morris *et al.*, 2012). As another example, the reconstructed genomes of ultra-small and uncultivated marine actinobacteria (“*Candidatus* Actinomarinidae”) were very small (<1‍ ‍Mb) and had a very low GC content of 33%‍ ‍(Ghai *et al.*, 2013). In addition, known obligate UMB of different lineages, such as “*Ca.* P. ubique” (*Alphaproteobacteria*), *Polynucleobacter* strains (*Betaproteobacteria*), and *A. minutum* and *R. lacicola* (*Actinobacteria*), showed similar “c-shaped” (curved-rod) cells (Table 1; *A. minutum* for Fig. 2; Hahn, 2003). This unique shape may be advantageous for the efficient acquisition of substances because of their increased surface-to-volume ratio of cells or grazing resistance against bacteriovorus protists for planktonic life in waters.

In contrast to aquatic environments, limited information is currently available on UMB, including the obligate type, from soil habitats. Janssen *et al.* (1997) previously reported anaerobic obligate UMB with very small ellipsoid to nearly spherical shapes (*e.g.*, *Opitutus* sp. VeCb1 with a cell volume of 0.030 μm^3^) belonging to the *Verrucomicrobiales* lineage from rice paddy soil using dilution culture techniques. Nakai *et al.* (2013) isolated and cultivated filterable strains from soil and sand suspensions; however, obligate UMB were not found among these strains. High-throughput sequencing of the 16S rRNA gene revealed that the smaller size fractions in soils were more likely to harbor rare or poorly characterized bacterial and archaeal taxa, such as *Acidobacteria*, *Gemmatimonadetes*, *Elusimicrobia*, *Verrucomicrobia*, and *Crenarchaeota* (Portillo *et al.*, 2013). However, further studies are needed to clarify whether the members detected in the small fractions contain UMB.

## Facultative UMB

Facultative UMB that contain a small proportion of larger cells with a cell volume >0.1 μm^3^ have not yet been characterized in detail (Table 1) because morphological changes throughout the growth cycle have only been examined in a limited number of UMB. *Endomicrobium proavitum* Rsa215 (now deposited as DSM29378^T^=JCM32103^T^) belonging to the phylum *Elusimicrobia* appears to be a well-studied example of facultative UMB. The phylum *Elusimicrobia* (former termite group 1 candidate phylum) was initially established with the cultivated ultramicrobacterium of *Elusimicrobium minutum* strain Pei191^T^ from the 0.2 μm-filtered filtrate—originally prepared as a growth promoting supplement for gut bacteria—of the gut homogenates of a scarab beetle larva (Geissinger *et al.*, 2009; Herlemann *et al.*, 2009). *E. proavitum* Rsa215 was isolated from the filtrate of the gut homogenate and was identified as a free-living bacterium of a novel class-level lineage in *Elusimicrobia* (Zheng *et al.*, 2016). *E. proavitum* has an unusual cell cycle that involves different cell forms, *i.e.*, cocci, rods, and budding-like cells, during the cell cycle. Under laboratory cultivation conditions, before growth commences, the cell population is comprised of a large population of UMB coccoid cells with a few rod-shaped cells (~3.5‍ ‍μm in length); small cocci are formed from a bud-like swelling at one pole of the rod-shaped cells during growth. Although its morphological variation in the host gut currently remains unclear, cell characteristics as observed in the laboratory result in the classification of facultative UMB. Another important trait for *E. proavitum* is the ability to fix nitrogen gas with a group IV nitrogenase, which was considered to harbor functions other than nitrogen fixation (Dos Santos *et al.*, 2012).

## Slender filamentous bacteria

In addition to ultramicrocells and UMB, slender filamentous bacteria have frequently been found in 0.2 μm-filtered fractions of environmental samples. Slender spirillum-shaped *Hylemonella gracilis* was isolated from filtrates of freshwater samples (*e.g.*, Hahn *et al.*, 2004; Nakai *et al.*, 2013) and passes through membrane filters with small pore sizes of not only 0.22–0.45 μm, but also 0.1 μm (Wang *et al.*, 2007). The smallest widths of *H. gracilis* cells are approximately 0.2 μm and close to filter pore sizes, which may allow its slender cells to “squeeze” through these pores. Regarding the quality control and assessment of filter sterilization, Wang *et al.* (2008) proposed that filterable slender bacteria, such as *H. gracilis* with small cell widths, may be used for the microbiological validation of membrane filters instead of *Brevundimonas diminuta*, which is the current standard strain tested.

**Fig. 3.**
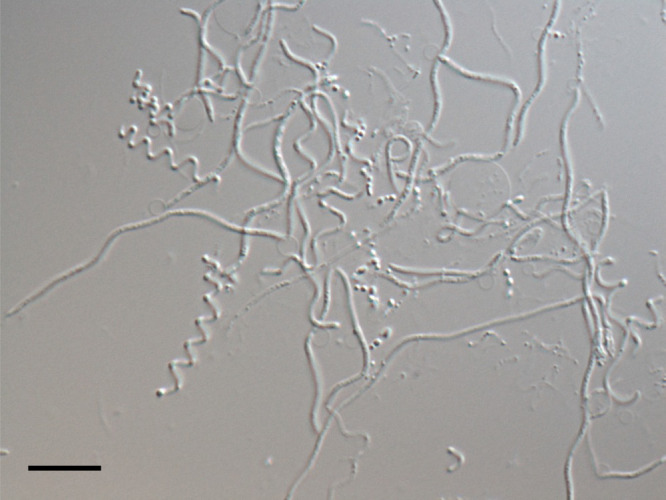
Micrograph of pleomorphic cells of *Oligoflexus tunisiensis* Shr3^T^. Cells were cultured in R2A medium for more than two weeks. This micrograph is slightly modified from the figure originally published in Nakai and Naganuma (2015). Scale bar: 10 μm.

During a screening of UMB, our group isolated a slender filamentous bacterium from the filtrate of a suspension of desert sands collected in Tunisia, and described *Oligoflexus tunisiensis* Shr3^T^, which represents the eighth novel class named *Oligoflexia* within the phylum *Proteobacteria* (Nakai *et al.*, 2014; 2016a). The cell shape of this species is mainly slender, filamentous, and of variable lengths, but shows a pleomorphism with other shapes, such as a spiral, spherical (or curled), or curved rod morphology (Fig. 3; Nakai and Naganuma, 2015). This polymorphic flexibility of cells with small widths down to 0.4 μm appears to be related to their ability to pass through membrane filters; however, it has not yet been clarified whether each morphological shape is associated with a resting state or other states. Regarding filamentous formation, this shape may be related to resistance to protozoan grazing, as reported in previous studies (*e.g.*, Jürgens *et al.*, 1999; Suzuki *et al.*, 2017a). The environmental sequences closely related (>97%) to the 16S rRNA gene sequence of *O. tunisiensis* were recovered from paddy soil, cyanobacterial bloom in lake water, bioreactors, and human skin using culture-independent approaches; however, their detection frequency was low, with at most ~0.6% (Nakai and Naganuma, 2015). Thus, *O. tunisiensis* and its relatives appear to be rare species, and their ecological roles are currently unclear; one possible role for *O. tunisiensis* may be incomplete denitrification to nitrous oxide, as inferred from its genome sequence (Nakai *et al.*, 2016a).

Despite the potential rarity of its occurrence, the size filtration method led to the isolation of an additional slender filamentous strain, *Silvanigrella aquatica* MWH-Nonnen-W8red^T^, with a pleomorphic morphology in the class (Hahn *et al.*, 2017). Hahn *et al.* (2017) reclassified the order *Bdellovibrionales*, including *Bdellovibrio* spp. known as small “bacteria-eating” bacteria (reviewed in Sockett, 2009), from the class *Deltaproteobacteria* to the class *Oligoflexia* based on in-depth phylogenetic analyses. Incidentally, 0.45-μm filtrates of environmental samples are frequently used for the enrichment culture of *Bdellovibrio* predatory bacteria. In the Genome Taxonomy Database (GTDB) based on genome phylogeny (https://gtdb.ecogenomic.org/; Parks *et al.*, 2018), the class *Oligoflexia* belongs to the candidate phylum “Bdellovibrionota”, named after the genus *Bdellovibrio*, and not the phylum *Proteobacteria*; its taxonomic assignment will be discussed in future studies. *Oligoflexia* very recently gained two more species, *Fluviispira multicolorata* 33A1-SZDP^T^ and *Silvanigrella paludirubra* SP-Ram-0.45-NSY-1^T^, from freshwater habitats (Pitt *et al.*, 2020). *Silvanigrella* spp. are phylogenetically closely aligned with “*Candidatus* Spirobacillus cienkowskii” (Pitt *et al.*, 2020), which is an uncultured pathogen of water fleas (*Daphnia* spp.) described morphologically almost 130 years ago (Metchnikoff, 1889). Since *Silvanigrella* spp. are isolated from the filtrates of micropore filtration, size fractionation may be an effective method for isolating the uncultivated pathogen as well as additionally overlooked agents in *Oligoflexia*. A detailed comparison within members of this class will also be important for pursuing the evolutionary acquisition and divergence of predatory and pathogenic behaviors.

## Diverse ultra-small members and their potentials

Metagenomic investigations on microbial communities have generated genomes for an astounding diversity of bacteria and archaea; CPR/Patescibacteria inhabiting groundwater has attracted increasing attention in recent years. Traditionally, certain types of groundwater bacteria were known to pass through a micropore filter (*e.g.*, Shirey and Bissonnette, 1991). Additionally, Miyoshi *et al.* (2005) phylogenetically characterized filterable microorganisms captured by 0.1-μm-pore-sized filters from deep aquifers of the Tono uranium mine, Japan and then discovered candidate divisions OD1 and OP11 (now recognized as candidate phyla “*Ca.* Parcubacteria” and “*Ca.* Microgenomates”, respectively) enriched by approximately 44% in 16S rRNA gene clones from the filtered fraction. The specific occurrence of “*Ca.* Parcubacteria” (OD1) in the 0.2-μm filtrate was also detected in deep-sea hydrothermal fluid (Naganuma *et al.*, 2007). It was previously unclear whether members of these candidate divisions were UMB. In subsequent studies using cryo-imaging, ultra-small cells (approximately 0.009±0.002 μm^3^) were reported in the filtrate of an aquifer water near Colorado, USA, which were enriched with the candidate divisions WWE3, OD1, and OP11, all recently belonging to CPR/Patescibacteria (Luef *et al.*, 2015).

Metagenomics was then used to reconstruct the genomes of filterable members in the aquifer system, representing >35 candidate phyla named CPR (Brown *et al.*, 2015). This highly diversified group of uncultivated bacteria may subdivide the domain *Bacteria* (Hug *et al.*, 2016); however, this scenario remains controversial (*e.g.*, Parks *et al.*, 2018; Zhu *et al.*, 2019). Importantly, measurements of replication rates (Brown *et al.*, 2016; Suzuki *et al.*, 2017b) and cryo-transmission electron microscopy images showing a dividing cell (Luef *et al.*, 2015) indicated that the extremely small cells of CPR/Patescibacteria are metabolically active and not simply ultramicrocells during starvation. Moreover, CPR/Patescibacteria genomes have been recovered from other environments, such as highly alkaline groundwater (Suzuki *et al.*, 2017b; Sato *et al.*, 2019), lakes (Vigneron *et al.*, 2019), soil (Starr *et al.*, 2018), and marine sediment (Orsi *et al.*, 2018) as well as the human microbiome (He *et al.*, 2015) and dolphin mouse (Dudek *et al.*, 2017), suggesting a wide distribution across environments. Besides describing ultra-small life forms with high phylogenetic novelty, genomic analyses of CPR/Patescibacteria members have provided information on their small genomes, fermentative metabolism, and other unusual features (*e.g.*, self-splicing introns varying in length and proteins encoded within their 16S rRNA genes; Brown *et al.*, 2015; Castelle *et al.*, 2018). Divergent 16S rRNA gene sequences prevent many specific phyla (*e.g.*, ~50% of “*Ca.* Microgenomates” [OP11] and 60% of candidate division WWE3) from being detected by typical PCR surveys with the universal bacterial primer set 515F and 806R (Brown *et al.*, 2016). The small genome sizes observed (often <1‍ ‍Mb) appear to be a reflection of a symbiotic lifestyle and/or high *in situ* selection pressure in a stable environment, rather than the genome streamlining of free-living obligate UMB, as described earlier, assuming streamlining characteristics (*e.g.*, highly conserved core genomes with few pseudogenes; Giovannoni *et al.*, 2014). Although the CPR/Patescibacteria genomes studied to date possess incomplete biosynthetic pathways for their cellular building blocks (*e.g.*, nucleotides and fatty acids; Castelle *et al.*, 2018), the possibility of their ability to *de novo* synthesize them by unknown pathways cannot be ruled out. Furthermore, their host-associated distribution was reported: “*Candidatus* Sonnebornia yantaiensis” of “*Ca.* Parcubacteria” (OD1) as an endoplasmic symbiont of the protist (Gong *et al.*, 2014) and TM7x bacterium of “*Ca.* Saccharibacteria” (TM7) attached to *Actinomyces odontolyticus* (He *et al.*, 2015), as shown in Table 1.

The features of small cell sizes and small genomes observed in CPR/Patescibacteria are shared by some members of the DPANN archaea, particularly *Nanoarchaeota* (Huber *et al.*, 2002), “*Ca.* Nanohalorchaeota” (Narasingarao *et al.*, 2012), and so-called ARMAN (archaeal Richmond Mine acidophilic nano-organisms; Baker *et al.*, 2010). DPANN including these UMA has been expanded by the addition of novel phylum-level groups, and, at the time of writing, encompasses at least ten different lineages (reviewed in Dombrowski *et al.*, 2019). In several cases, except for the members of “*Ca.* Nanohalorchaeota”, as with CPR/Patescibacteria, DPANN-affiliated UMA showed an ectosymbiotic localization: *Nanoarchaeum equitans* attached to *Ignicoccus hospitalis* (Huber *et al.*, 2002), “*Ca.* Nanopusillus acidilobi” and its host *Acidilobus* species (Wurch *et al.*, 2016), and “*Ca.* Mancarchaeum acidiphilum” Mia14 (ARMAN-2-related organism) and its host *Cuniculiplasma divulgatum* (Golyshina *et al.*, 2017) (other data in Table 1). Additionally, DPANN organisms lack the ability to biosynthesize their building blocks (Castelle *et al.*, 2018). Although it is still unclear whether these symbiotic or parasitic lifestyles represent a way of life for the CPR/Patescibacteria and DPANN groups, the cases described above indicate that several members of these groups appear to be important in organism-organism interactions.

**Fig. 4.**
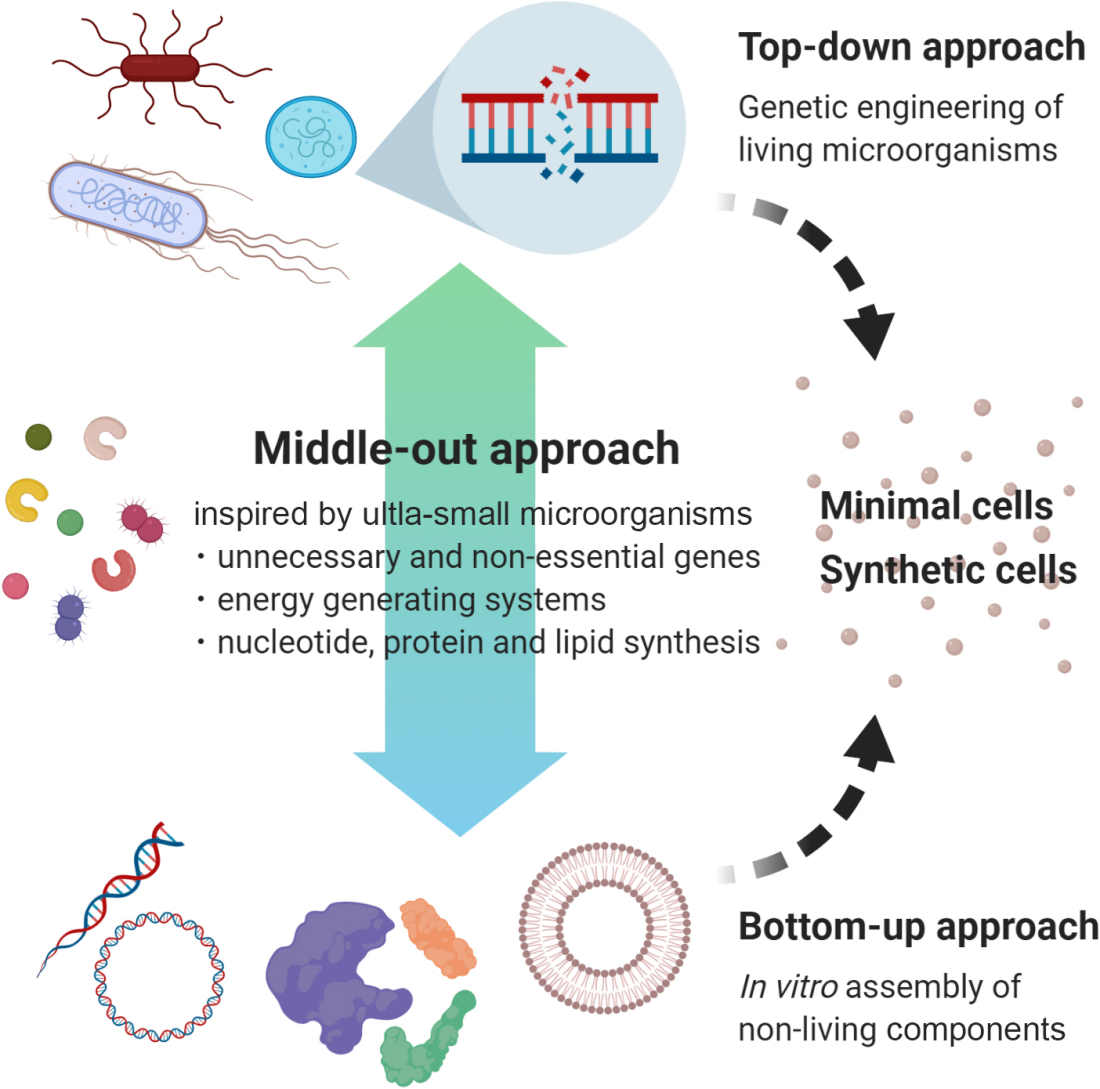
A schematic diagram of the “middle-out” approach toward the development of minimal cells or synthetic cells. This approach, inspired by the unusual biology of ultra-small life forms, may provide a new perspective to traditional top-down or bottom-up approaches. This figure was created with BioRender (https://biorender.com/).

The characterization of ultra-small life forms may provide a new perspective for minimal cells and synthetic cells. In the field of synthetic biology, the top-down approach has been employed to reduce and simplify the genomes of microbial cells by genetic engineering, and then to identify essential genes for living systems; the bottom-up approach, which is the opposite of the top-down approach, has been used to examine what is sufficient for living systems by assembling non-living components, such as nucleic acids, proteins, and lipids (*e.g.*, Matsuura *et al.*, 2011; Xu *et al.*, 2016). In this context, DeWall and Cheng (2011) pointed out that the small genomes of microorganisms in nature may be models for the identification of a minimal genome. Since the ultra-small members described here as well as free-living obligate UMB already harbor small and sometimes streamlined genome structures (<2‍ ‍Mb) through the loss of unnecessary components, the “middle-out” approach, referring to the metabolic pathway of these members (Fig. 4), which effectively combines traditional top-down and bottom-up approaches, will be useful for the rational design of artificial cells.

## Conclusions

Numerous cultivation efforts have clearly shown that some previously uncultured members remain viable in small-size fractions. Some obligate UMB are ubiquitous and dominant in water systems and may play important roles in natural microbiome functions. In parallel, the advent of high-throughput sequencing technology has greatly expanded our knowledge of ultra-small microbial diversity. Future studies are required to shed light on small microorganisms hidden in various environmental samples (*e.g.*, soils and sediments) other than aqueous environments, and on the ecophysiological traits and biogeochemical roles of these members, including CPR/Patescibacteria and DPANN. Further studies on “extreme” microorganisms at the lower size limit will undoubtedly lead to new conundrums about life on Earth.

## Citation

Nakai, R. (2020) Size Matters: Ultra-small and Filterable Microorganisms in the Environment. *Microbes Environ ***35: **ME20025.

https://doi.org/10.1264/jsme2.ME20025

## Figures and Tables

**Table 1.** An overview of ultra-small and filterable microorganisms in the environment

Taxa	Phylum (and class for *Proteobacteria*)	Isolation source	Cell shape	Cell size (length×width and/or volume)	Genome size (Mbp)	Physiological and ecological trait(s) or its potential	Reference
Ultramicrocells							
*Staphylococcus aureus* 8325-4	*Firmicutes*	derivative of *S. aureus* NCTC8325 (patient’s strain)	cocci	cell size reduction from 0.69±0.08 to 0.41±0.08 μm	n.d.	host cell invasion, starvation-associated cell size reduction	Watson *et al.* (1998)
*Pseudomonas syringae* pv. *syringae* B728a	*Proteobacteria* (*γ-proteobacteria*)	snap bean leaflet	rods	cell length reduction from ~2.5 to ~1.2 μm	6.09	host cell invasion, leaf environment-induced cell size reduction	Monier and Lindow (2003); Feil *et al.* (2005)
Obligate ultramicrobacteria and related candidates							
“*Candidatus* Pelagibacter ubique” HTCC1062	*Proteobacteria* (*α-proteobacteria*)	coastal sea	curved rods	0.01 μm^3^	1.31	glycine auxotrophy, rhodopsin-based photometabolism, utilization of one-carbon compounds	Rappé *et al.* (2002); Tripp (2013); Giovannoni (2017)
“*Candidatus* Fonsibacter ubiquis” LSUCC0530	*Proteobacteria* (*α-proteobacteria*)	coastal lagoon	curved rods	1.0×0.1 μm	1.16	glycine auxotrophy, rhodopsin-based photometabolism, tetrahydrafolate metabolism**	Henson *et al.* (2018)
*Sphingopyxis alaskensis* RB2256	*Proteobacteria* (*α-proteobacteria*)	fjord estuary	short rods	0.05–0.09 μm^3^	3.35	utilization of various amino acids, resistance to heat shock, H_2_O_2_, and ethanol	Eguchi *et al.* (1996); Schut *et al.* (1997)
*Aurantimicrobium minutum* KNC^T^	*Actinobacteria*	freshwater river	curved rods	0.7–0.8×0.3 μm; 0.04–0.05 μm^3^	1.62	rhodopsin-based photometabolism**	Nakai *et al.* (2015, 2016b)
*Rhodoluna lacicola* MWH-Ta8^T^	*Actinobacteria*	freshwater lake	curved rods	0.85×0.30 μm; 0.053 μm^3^	1.43	rhodopsin-based photometabolism	Hahn *et al.* (2014); Keffer *et al.* (2015)
*Rhodoluna limnophila* 27D-LEPI^T^	*Actinobacteria*	freshwater pond	short rods	0.49×0.28 μm	1.40	nitrate uptake and nitrite excretion system**	Pitt *et al.* (2019)
“*Candidatus* Planktophila rubra” IMCC25003	*Actinobacteria*	freshwater lake	curved rods	0.041 μm^3^	1.35	catalase-dependent growth	Kim *et al.* (2019)
“*Candidatus* Planktophila aquatilis” IMCC26103	*Actinobacteria*	freshwater lake	curved rods	0.061 μm^3^	1.46	catalase-dependent growth	Kim *et al.* (2019)
*Polynucleobacter necessarius* subsp. *asymbioticus* QLW-P1DMWA-1^T^	*Proteobacteria* (*β-proteobacteria*)	freshwater pond	straight rods	0.7–1.2×0.4–0.5 μm	2.16	utilization of low-molecular-weight substrates	Hahn *et al.* (2012); Meincke *et al.* (2012)
*Opitutus* sp. VeCb1	*Verrucomicrobia*	rice paddy soil	ellipsoids	0.49×0.33 μm; 0.030 μm^3^	n.d.	utilization of sugars and sugar polymers, strict fermentative metabolism, oxygen tolerance	Janssen *et al.* (1997); Chin *et al.* (2001)
Facultative ultramicrobacteria							
*Endomicrobium proavitum* Rsa215	*Elusimicrobia*	gut homogenate of *Reticulitermes santonensis*	cocci, rods showing budding cell division	0.3–0.5 μm (for cocci); 0.5–3.5×0.15–0.30 μm (for rods)	1.59	nitrogen fixation	Zheng and Brune (2015); Zheng *et al.* (2016)
*Chryseobacterium solincola* NF4	*Bacteroidetes*	lake sediment	cocci, rods showing budding cell division or cell septation	0.004–0.04 μm^3^ (for cocci); 0.1–0.3 μm^3^ (for rods)	~1.7	ectoparasite of *Bacillus subtilis*	Suzina *et al.* (2011); Duda *et al.* (2012)
Slender filamentous bacteria							
*Hylemonella gracilis* CB	*Proteobacteria* (*β-proteobacteria*)	freshwater	spirals	0.12 μm^3 ^(smallest width=0.2 μm)	n.d.	n.d.	Wang *et al.* (2007, 2008)
*Oligoflexus tunisiensis* Shr3^T^	*Proteobacteria* (*Oligoflexia*)*	desert sand	pleomorphic (rods, filaments, spirals, and spherical [or curled] cells)	various lengths×0.4–0.8 μm (for filaments)	7.57	multidrug resistance, incomplete denitrification**	Nakai *et al.* (2014, 2016a)
*Silvanigrella aquatica* MWH-Nonnen-W8red^T^	*Proteobacteria* (*Oligoflexia*)*	freshwater lake	pleomorphic (rods, filaments, and spirals)	3.6×0.6 μm (for rods)	3.51	antimicrobial peptides, plasmid-encoded type IV secretion systems**	Hahn *et al.* (2017)
*Silvanigrella paludirubra* SP-Ram-0.45-NSY-1^T^	*Proteobacteria* (*Oligoflexia*)*	freshwater pond	pleomorphic (rods and filaments)	various lengths	3.94	utilization of limited substrates	Pitt *et al.* (2020)
*Fluviispira multicolorata* 33A1-SZDP^T^	*Proteobacteria* (*Oligoflexia*)*	freshwater creek	pleomorphic (rods and filaments)	various lengths	3.39	violacein-like production	Pitt *et al.* (2020)
CPR/Patescibacteria bacteria							
WWE3-OP11-OD1 bacteria	candidate division WWE3, “*Candidatus* Microgenomates” (OP11), “*Candidatus* Parcubacteria” (OD1)	deep aquifer	cocci or oval-shaped	0.009±0.002 μm^3^	0.69–1.05	potential interaction with other bacterial cells via pili-like structures	Luef *et al.* (2015)
“*Candidatus* Sonnebornia yantaiensis”	“*Candidatus* Parcubacteria” (OD1)	ciliated protist *Paramecium bursaria*	straight rods	1.6–1.9×0.5–0.6 μm	n.d.	endoplasmic symbiont of the ciliate *P. bursaria*	Gong *et al.* (2014)
TM7x bacterium	“*Candidatus* Saccharibacteria” (TM7)	human oral cavity	cocci	0.2–0.3 μm	0.71	ectosymbiont of *Actinomyces odontolyticus*	He *et al.* (2015)
DPANN archaea							
*Nanoarchaeum equitans*	*Nanoarchaeota*	submarine hot vent	cocci	0.4 μm	~0.5	ectosymbiont of *Ignicoccus hospitalis*	Huber *et al.* (2002)
“*Candidatus* Nanopusillus acidilobi”	*Nanoarchaeota*	hot spring	cocci	0.1–0.3 μm	0.61	ectosymbiont of *Acidilobus* species	Wurch *et al.* (2016)
“*Candidatus* Nanoclepta minutus” Ncl-1	*Nanoarchaeota*	hot spring	flagellated cocci	~0.2 μm	0.58	ectosymbiont of *Zestosphaera tikiterensis*	John *et al.* (2019)
“*Candidatus* Nanosalina” sp. J07AB43	“*Candidatus* Nanohaloarchaeota”	hypersaline lake	cocci-like	0.6 μm	1.23	possible free-living lifestyle	Narasingarao *et al.* (2012)
“*Candidatus* Nanosalinarum” sp. J07AB56	“*Candidatus* Nanohaloarchaeota”	hypersaline lake	cocci-like	0.6 μm	1.22	possible free-living lifestyle	Narasingarao *et al.* (2012)
ARMAN-2, -4, and -5	“*Candidatus* Micrarchaeota”	acid mine drainage	cocci	~0.5 μm	~1.0	potential interaction with *Thermoplasmatales* cells via pili-like structures	Baker *et al.* (2010)
“*Candidatus* Mancarchaeum acidiphilum” Mia14	“*Candidatus* Micrarchaeota”	acid mine drainage	n.d.	n.d.	0.95	ectoparasite of *Cuniculiplasma divulgatum*	Golyshina *et al.* (2017)

n.d.: no data. * The proteobacterial class *Oligoflexia* is classified in the candidate phylum “Bdellovibrionota” in the Genome Taxonomy Database (GTDB). ** Putative physiological traits are inferred from their genomic and plasmid annotation.
